# Cases of Tetatnus, with Observations upon the Disease; in Continuation

**Published:** 1809-12

**Authors:** John Howship

**Affiliations:** Surgeon


### *Cases of Tetatnus, with Observations upon the Disease; in Continuation; by* John Howship, Surgeon.

*A Case of* Lock-Jaw, *from the Irritation of an extensive Wound, in which Opium certainly checked the Progress of the Disease, and probably saved the Life of the Patient.*

THOMAS PLATT, forty-six years of age, seaman on board of his Majesty's ship Bellerophon, was knocked down in the great naval engagement that took place upon the 21st of October, 1805. He was struck by a grape-shot of about one pound weight, which produced a compound fracture of the thigh. This man was laid in the cockpit, together with many, till his turn came for being dressed, when the proper applications were made to the wounded parts, consisting of very light dressings, supported by a linen roller.

For the first day or two, this, like the majority of gun-shot wounds, was productive of but little pain. The wound however, being complicated by shattered fragments of a broken bone, a high degree of inflammatory irritation soon commenced, which in all probability would not have occurred, had the accident been produced by a ball of sufficient magnitude to have struck off the limb at once.

Upon the third day he was brought on shore, and placed upon the very comfortable establishment that had been prepared for the accommodation of the wounded sailors, in the naval hospital at Gibraltar. At this time there was considerable heat, pain, and tension about the wound; and this for some days afterwards continued to increase, notwithstanding the diligent use of fomentation and poultice. His general health however was good, and as his appetite remained unimpaired, his strength was very sufficiently supported by a sound nourishing diet.

At the close of the second week he was doing well in most respects, but the pain and inflammation in the wounded limb were very considerable, the discharge of matter also was now very great; and this latter source of exhaustion, during the progress of the third week, became every day of more importance; but towards the latter end of the third week the inflammatory symptoms became more moderate; still, however, he was deprived of his rest at night; for although the excessive heat and throbbing in the wound had subsided very much, still the sharp stitches

 and and shooting pain, to which he had more or less been subject from the first, continued to harrass him.

Upon the 22d day the irritated condition of the limb was becoming more and more severe; the twitches of sharp pain were now so frequent, that the thigh was in a state of constant and distressing pain, subjected to occasional aggravation, as often as the wounded and lacerated muscles were thrown into contraction upon the sharp and splintered portions of the fractured bone.

During the forenoon of this day, he first expressed his surprise, at finding that something ailed his mouth and jaws. This affection he described as perfectly painless; and the only observation he made upon it was, that it prevented him from opening his mouth so freely as usual, and that together with this, he found that he could not swallow "cleverly."

The discharge from the wounds was at this time manifestly less profuse than it had been for some time before. The pain and spasmodic action of the muscles of the thigh were the same to which he had all along been subject; these always commenced among the wounded parts, and passed upwards towards the body; never however extending their effects beyond the limb, or producing any painful spasms in the muscles of the trunk.

From eight in the morning he took two grains of calomel and the same quantity of opium in a pill, repeated every hour till noon, when he was ordered four grains of calomel and three of opium, every hour; which plan of treatment was continued through the following night, and upon the 23d day he found himself much better. He said he could open his mouth more freely, besides which, he was able to swallow with less difficulty than before. He observed that he rarely now felt the starts in his thigh, and it was evident that he was materially, and indeed very generally, relieved by the opium. During this day and night the treatment of the day before was continued. On the 24th day, at eight in the morning, this man's symptoms being greatly relieved, four grains of calomel with three of opium, were ordered to be taken every second hour. He passed the day and the night following very comfortably. From the time of his having commenced with the opium, there had been no action in the bowels; two laxative injections, therefore, were this day thrown up, and succeeded in clearing the rectum of its contents. The appetite still remained as keen as ever.

Twenty-fifth day, much better, and but very rarely disturbed turbed by the shooting pains darting through the thigh; besides which, he found very little remaining stiffness about the jaws. The calomel and opium, of each two grains, were ordered to be given every second hour.

Upon the 26th day, the quantity that he took of opium was still farther diminished, by his pill being ordered every third, instead of every second hour.

In this way he continued to take opium in diminishing quantity for some days: and before the remedy was laid aside altogether, he was most completely relieved from the inconvenience he had experienced in swallowing, and from the stiffness that had prevented him from opening his mouth; added to which, the uneasiness felt in the limb itself, was much less than at any time since the wound had been received.

It was in the above case interesting to observe, that notwithstanding the large proportion of opium taken, the patient was, during the period of its exhibition, on the average, more watchful than a person in health usually is, although by his answers, and the account which, from time to time, he gave of himself, it was evident enough that he was not kept awake, indeed scarcely disturbed, by the recurrence of the spasms in the wounded limb.

The remedy, in this instance, seemed to have exerted its powers in the most happy and satisfactory manner. It succeeded most decidedly in allaying effectually the morbid irritation that had been proved to exist in the nervous system; while at the same time it did not produce the least apparent effect upon the brain. His conversation in the day and in the night, was always calm and clear, but never influenced by the least perceptible degree of the dull and lethargic state which usually is produced by the use of opium. His pulse never was altered from its natural state by the treatment adopted, the only trifling inconvenience sustained was the disposition it gave to a costive state of the bowels.

The consideration of the case last described, in its relation to those other examples of tetanus that have been detailed, seems to suggest that there certainly must be a great variety in the intensity of predisposition to these complaints. This is not in itself a new fact, although the accurate history of individual cases must still greatly tend to illustrate the very extensive application of which it is capable.

In the case of Platt, even the recurrence of the spasms, and the severe pain in the wounded limb, did not ever induce induce the same description of painful spasm in the muscles of the jaws, &c. which in the progress of most of the other cases took place towards the establishment of the disease. Neither did the act of deglutition even produce to him any sort of pain, much less did it prove to be the commencement of extended and severe torment, such as very generally arises in tetanus; the dread of which torments has determined many an unhappy sufferer in this disease, never to make a second attempt to swallow either food or medicine.

There may be many shades of strength in the constitutional predisposition to these complaints; and in all probability it may depend very much, or perhaps entirely, upon the peculiar state of this predisposition, and not at all upon the mere strength, or degree of tone in the contractile fibre, whether an irritation from a wound shall, in exerting its full effect upon the brain and nerves, produce an aggravated and rapidly advancing state of tetanus, eventually fatal; or only spasms in the limb or part wounded; the convulsive action extending itself upwards towards the brain, with consequent stiffening of the parts about the jaws; or, as is infinitely more often the case, startings and catchings of a spasmodic nature, being altogether confined to the situation of the original injury.

Upon the same principle it may be explained, why a remote irritation being removed, shall in one individual be obviously the means of saving life; the existing morbid state of the nerves not being sufficiently great to keep up the effect, after the removal of the exciting cause: while in another case, perhaps one of equal at the commencement, the removal of the primary irritation shall do no more than produce a manifest change in the ground, as it were, upon which the continuing disease rests; the operation for the removal of the exciting cause shall in this way be attended with material relief, considering the case in one point of view; while seen in another, it affords little or no foundation for any hope of recovery.

The peculiar and unknown condition of the nervous matter may in this disease be such, that the comparatively small proportion of nervous energy required for the continuing of the involuntary and vital actions of the system, may become altogether vitiated; or the influence of the whole nervous system may finally sink from its mutual and necessary relationship to the voluntary and involuntary contractile strictures in the body; and the harmonious unison of action prevailing amid the several systems that exist exist together in the animal machine, be thus lost for ever.

In all the various gradations of tetanus, the disease has still the same distinguishing characters, rendering it perfectly unlike to any other complaint whatever. In epilepsy the voluntary muscular parts of the body are thrown into a state of contraction, but the impressions producing action are not permanent; they are excessive in point of force, as is proved by the undue degree of intensity of action in the contractile fibre; the impressions however are to a certain degree transitory; they may succeed each other rapidly, but during the space of time or interval, that occurs between every preceding and the next following state of excitement, the muscular parts may certainly be considered to be left in a state of relaxation.

If that very peculiar and highly mysterious condition of the nervous system, in which catalepsy takes place, be duly considered, it will appear that there is evidently a state of action in the voluntary muscles of the body; yet although action in voluntary parts, still the action at that particular time is independent of volition. This, however, is by no means to be considered as an action resulting from any nervous impression of too great an intensity, for experiment has repeatedly evinced, that during the influence of this curious affection upon the body, the position of the limbs may, by a very moderate power, be altered at pleasure; although, while the body is allowed to remain undisturbed, the posture of every part remains unchanged for whatever length of time the disease may remain.

In this case there cannot be supposed to be a succession of strong and violent impressions conveyed from the sensorium through the nerves to the muscular parts of the system. It is a permanent but mild and gentle influence, an impression, that like a gentle current pervades and passes onward into all the muscular structure, through the nervous matter of the system. The affection principally rests upon all the moving parts of the body subject to volition; and from the moderate force of the impression conveyed, it bears some analogy to the limited nature of every voluntary effort. A clear line of distinction, however, is the following, that the most persevering effort of volition, has not the power of continuing any voluntary muscle in a state of permanent contraction for any length of time; the muscular parts are found to fail in their obedience, dience, the fibres soon tremble, and finally relax themselves altogether.

It is in Tetanus alone that a state of permanent, and at the same time, intensely violent contraction takes place in the voluntary system of muscles. This permanency of contraction, however, does not even in this complaint ever exist for any great length of time, when the severity of action is exquisitely formed. It would seem that the contractile power resident in every muscular part, is in its nature incapable of sustaining a continued state of the most violent contraction, beyond a very short space of time. In tetanus, it is true, that while the more severe attacks of spasm, pervading equally every part of the trunk and extremities, are observed to continue in full force for a few minutes only, afterward relaxing, the muscles of the jaws are still so affected, as to keep the teeth in perfect, perpetual, and obstinate contact; yet even here it may be observed, that there is by no means, at all times, the same degree of intensity in the contraction of the muscular parts. It will always be found, that in the moments of the more aggravated sufferings, the teeth are clenched more closely and firmly together than they were before; and during the intervals of time that elapse between the attacks of universal spasm, the jaws may in some instances, be in some degree separated by the dexterous and careful introduction of a lever well adapted to the purpose.

The manner in which the intellect is either altogether abolished, held in partial restraint, or left in a state of perfect freedom, is subject to great variety, in the various disorders to which muscular action is exposed.

In Epilepsy the consciousness is lost altogether, and this is a provision dispensed in mercy, by the agency of which the unhappy sufferer is rescued from that sense of pain, which, from the convulsed state of the voluntary muscles of the system must otherwise have proved a burthen almost too heavy for humanity to support. After the fit of epilepsy is over, the senses gradually return, and the person awakes as from a dream; and it is remarkable, that although the memory may have retained a clear apprehension of the succession of past incidents up to the moment when the fit commenced, yet the time spent in the convulsion, when regarded by the mind's eye, is considered as a void, a space that cannot be rendered an object of perception.

In catalepsy again, the condition in which the intellect is involved is widely different.

In epilepsy the action of every faculty and sense is completely suspended, superceded, and, for the time, perfectly terminated; but in catalepsy the fancy has been generally described as upon the wing, and sometimes it has been engaged in scenes, the descriptions of which are greatly calculated to excite the highest admiration and astonishment in a reflecting mind, while they certainly evince that we have not a more limited acquaintance with any of the works of creation than we have with ourselves.

There are some well authenticated histories of catalepsy in which the mind, or fancy, has been described to have been busily occupied as in a reverie, but incapable of receiving any impression whatever from without; while, on the other hand, cases have occurred, in which, notwithstanding all power of action has been withheld, the conciousness of existence and life, together with the sense of hearing, have been certainly retained; in this unfortunate situation the person has been a silent witness to the whole of the preparations for the funeral, without the power of urging a single muscle into action, until, at last, the strong conviction of impending horror pressed most heavily, and, in the moment when the top of the coffin was to have been screwed down, the supposed corpse has, by a desperate effort of volition, succeeded in making a visible token of animation, and has afterwards perfectly recovered.

In tetanus the sense of sight remains while the eye is fixed and stareing, perfectly beyond the controul of voluntary motion. The limbs are held fast in spasm, while the mind is feelingly alive to all the aggravated sufferings of the body. In the most intolerable fits of pain and spasm, the senses of hearing, seeing, and smelling, remain perfect and clear, and the patient perceives and apprehends readily the meaning of every word that passes at the bedside.

Although for the moment he is actually deprived of all power of expression, he will, after the attack has passed over, express correctly enough any opinion that might have been during the fit suggested upon his situation, on the probability of his recovery.

In Epilepsy, the state of the brain may be said to be tolerably well ascertained. There is every symptom indicating preternatural fulness of vessels within the head. There is a state of total oblivion in all that relates to mind during during the fit; and the first expression upon the return of sense is very generally a complaint of severe pain in the head, precisely the same feeling of general uneasiness in the brain, which results from the application of any other cause of temporary pressure to the sensorium.

In Catalepsy there is every reason to consider that the energies of the brain are not incommoded by the least approach to a state of pressure. There are, in this case, many objections to any supposition of local fulness of the vessels within the head, particularly the following, that in catalepsy the circulating system acts with so strong a degree of languor, that the pulse can no longer be perceived, either at the wrist, or in any other part of the body. The fancy, in this complaint, is in general very particularly active, and the system is in a state of obvious repose, subsisting in perfect tranquillity, undisturbed by any tendency to spasm, general or particular.

In Tetanus, also, there is not the least evidence of pressure upon the brain, nor is there any proof, nor any reason to suspect a suspension of its energies during the attacks of the most violent spasm. In this disease the variations that are daily arising in the features of the complaint may, by attention, be readily ascertained as they occur at the bedside; but it will be seen that throughout, the mind still continues to stand perfectly uninfluenced by the most aggravated severity of the disorder. Never is there complaint of pain, weight, or lightness of the head, nor indeed of any affection whatever of the brain, even for a moment, notwithstanding that the senses are sometimes, as it were, absolutely transfixed within their very porches.

( To be continued. )

